# In Vitro Evaluation of the Antimicrobial Activity of *Origanum vulgare* Essential Oil Against ESBL-Producing Strains of *Escherichia coli* and *Klebsiella pneumoniae*

**DOI:** 10.3390/cimb48040373

**Published:** 2026-04-03

**Authors:** Andrea González Carrión, Mónica Espadero Bermeo, Inés Malo Cevallos, Ronny Ricardo Alejandro

**Affiliations:** 1 Universidad Politécnica Salesiana, Cuenca 010102, Ecuador; angy2003gc@outlook.com (A.G.C.); mespadero@ups.edu.ec (M.E.B.); 2 National Institute of Public Health Research (INSPI), Center of Reference Antimicrobial Resistance (CR-RAM), Cuenca 010107, Ecuador; ralejandro@inspi.gob.ec

**Keywords:** essential oil, antimicrobial activity, ESBL, *Klebsiella pneumoniae*, *Escherichia coli*, cefepime, synergism

## Abstract

One of the greatest threats to global public health is antimicrobial resistance (AMR), due to the increasing number of infections caused by extended-spectrum β-lactamase (ESBL)-producing Enterobacteriaceae. *Escherichia coli* and *Klebsiella pneumoniae* ESBL-producing bacteria possess resistance mechanisms that inactivate β-lactam antibiotics by hydrolyzing their β-lactam ring, thereby limiting conventional therapeutic options. In response to this problem, the objective of this exploratory in vitro study was to evaluate the antimicrobial activity of *Origanum vulgare* (oregano) essential oil and its interaction with the antibiotic cefepime using in vitro methods. Antimicrobial susceptibility tests were performed, including determination of the minimum inhibitory concentration by the microdilution method with statistical analysis, and evaluation of the fractional inhibitory concentration index using the checkerboard method. In addition, advanced methods such as MALDI-TOF mass spectrometry for bacterial identification and PCR were employed for the identification of resistance genes. The studied strains exhibited both phenotypic and genotypic resistance. The MIC of the essential oil was 1024 µg/mL for ESBL-producing *E. coli* and 2048 µg/mL for ESBL-producing *K. pneumoniae*, whereas the ATCC strains showed higher susceptibility. The FICI values indicated synergism in *E. coli* (FICI = 0.188) and an additive effect in *K. pneumoniae* (FICI = 0.563). Oregano essential oil exhibits antimicrobial activity and the ability to potentiate the effect of cefepime, suggesting its potential as a therapeutic adjuvant. Additional studies are required, including a larger number of strains, cytotoxicity analyses, and clinical validation.

## 1. Introduction

Antimicrobial resistance (AMR) is one of the main challenges for public health because it compromises the effectiveness of antibiotic treatments [1], particularly due to the increasing prevalence of extended-spectrum β-lactamase (ESBL)-producing Enterobacteriaceae, which inactivate β-lactam antibiotics such as penicillins and cephalosporins. These β-lactamase enzymes are encoded by acquired genes or variants such as: *bla_CTX-M*, *bla_TEM* and *bla_SHV*, which confer resistance to β-lactam antibiotics [2], thereby limiting therapeutic options and increasing morbidity and mortality associated with infections. The World Health Organization (WHO) classifies ESBL-producing bacteria as priority pathogens for the development of new treatments, and reports indicate that 60% of nosocomial infections in Latin America are caused by β-lactam-resistant Enterobacteriaceae [3].

*Escherichia coli* is an opportunistic bacterium associated with cases of cystitis, sepsis, and pneumonia due to the acquisition of resistance genes through mobile genetic elements (plasmids) [4,5]. *Klebsiella pneumoniae* also acquires ESBL production and is a nosocomial pathogen associated with approximately 50% of bacteremia cases, as well as a high capacity for resistance to multiple antibiotics [6].

Medicinal plants have been valued for their therapeutic properties, playing a fundamental role in traditional medicine. This practice has been integrated into modern medicine through the isolation and characterization of phytochemicals or bioactive compounds such as terpenoids, phenolic compounds, glycosides, and alkaloids, which exhibit anti-inflammatory, antimicrobial, and antifungal effects [7,8].

Among the methods used for the extraction of bioactive compounds are non-conventional techniques such as ultrasound-assisted extraction and supercritical fluid extraction, as well as conventional methods including maceration, percolation, decoction, solvent extraction, and steam distillation [9]. The latter is particularly useful because it yields essential oils containing bioactive components with therapeutic potential [10].

The essential oil of *Origanum vulgare* (oregano), belonging to the Lamiaceae family, is rich in phenolic monoterpenes and phenylpropanoids, with carvacrol and thymol being the main compounds responsible for its antimicrobial activity [11]. Studies by Nurzyńska demonstrate that essential oils exert inhibitory effects on the growth of both Gram-positive and Gram-negative bacteria, with particular emphasis on *Escherichia coli* and *Klebsiella pneumoniae*, due to the presence of metabolites in oregano essential oil that act through multiple mechanisms, including destabilization of the outer membrane, suppression of resistance gene expression, and inhibition of protein synthesis. In light of this emergency, oregano essential oil has been considered a natural adjuvant [12].

Carvacrol has demonstrated synergistic effects with β-lactam antibiotics, reversing resistance to cephalosporins in resistant *E. coli* strains [13]. Si et al. reported minimum inhibitory concentrations (MIC) of *Origanum vulgare* essential oil of 0.5 µg/mL and 0.8 µg/mL against ESBL-producing *E. coli* strains, in addition to a synergistic effect with fluoroquinolones or doxycycline, which reduced the standard dose of these antibiotics by up to eightfold [14].

## 2. Materials and Methods

### 2.1. Bacterial Strains

Four bacterial strains were used, provided by the National Institute of Public Health Research (INSPI, Cuenca, Ecuador) due to their epidemiological relevance regarding resistance mechanisms: *E. coli* ATCC 25922 and *K. pneumoniae* ATCC 700603, employed as quality control strains, and two clinical bacterial isolates (uropathogens) that produce ESBL: *E. coli* (ID 323) and *K. pneumoniae* (ID 132). The strains were preserved at −20 °C in cryovials with glycerol and reactivated in BHI. For each assay, fresh cultures (≤24 h) were used to ensure viability.

### 2.2. Identification by MALDI-TOF MS

Taxonomic identification of the study strains was performed by MALDI-TOF mass spectrometry at the MEDICULT laboratory (Cuenca, Ecuador). Fresh colonies (18 h) were taken from blood agar plates and placed onto a polished steel target plate for identification using the Microflex LT system (Bruker Daltonics, Bremen, Germany). The generated protein spectrum was compared with a reference database, providing score values, where scores ≥ 2.0 indicated reliable identification at the genus and species levels.

### 2.3. Susceptibility Analysis for Phenotypic Detection

Phenotypic detection of ESBL production was carried out using the double-disk synergy test described by the Jalier technique [15], employing the antibiotics amoxicillin/clavulanic acid (20/10 µg), aztreonam (30 µg), cefotaxime (30 µg), ceftazidime (30 µg), and cefepime (30 µg).

Mueller–Hinton agar plates were inoculated with a bacterial suspension adjusted to the 0.5 McFarland standard. The disks were placed at a distance of 25 mm and incubated at 37 °C for 18–24 h. The appearance of deformation or enlargement of the inhibition zone toward the amoxicillin/clavulanic acid disk was considered indicative of ESBL production. Inhibition zone diameters were measured and interpreted according to CLSI breakpoints [16].

### 2.4. Molecular Identification by PCR

Five to six fresh colonies from each strain (the two clinical strains and the *E. coli* ATCC 25922 strain were used as a negative control, and the *K. pneumoniae* ATCC 700603 strain was used as a positive control for the *bla_SHV* gene) were collected and placed in 1 mL of TAE buffer, then incubated in a Thermomixer (Senacyt, Cuenca, Ecuador) at 95 °C with constant agitation for 10 min, followed by centrifugation to mechanically extract the genetic material. PCR reactions were prepared in a final volume of 24 µL containing 10 µL of molecular-grade water, 12 µL of GoTaq^®^ Master Mix (Promega Corporation, Madison, WI, USA), 0.5 µL of forward primer, 0.5 µL of reverse primer, and 1 µL of DNA, more details are provided in Table 1. Amplification was performed for two hours over 36 cycles in a Bio-Rad thermocycler (Hercules, CA, USA), following the conditions in Table 1. The samples were loaded onto a 1% agarose gel prepared with 1% TAE buffer and SafeView Classic^®^ (Applied Biological Materials, Richmond, BC, Canada) as the staining agent, and electrophoresis was carried out at 80 V for 40 min. After electrophoresis, the gel was visualized using a Bio-Rad gel documentation system for gene detection.

### 2.5. Origanum vulgare Essential Oil

The essential oil was acquired from the company KHIWA NATURAL (Quito, Ecuador), obtained by steam distillation, and accompanied by a certificate reporting the purity of the essential oil, density (0.935 g/mL). The quantified content of secondary metabolites was carried out by the GC-MS technique, detailed in Table 2.

### 2.6. Determination of the Minimum Inhibitory Concentration (MIC)

The MIC of *Origanum vulgare* essential oil and the antibiotic cefepime was determined using the broth microdilution method in 96-well plates, following CLSI M100 guidelines [16].

Two-fold serial dilutions of the essential oil were prepared in Mueller–Hinton Broth (MHB). For *K. pneumoniae* 132, the tested range was 4 to 2048 µg/mL, while for the remaining strains, the range was 2 to 1024 µg/mL. Each well was inoculated with a final bacterial concentration of 5 × 10^5^ CFU/mL. DMSO at a final concentration of 1% (*v*/*v*) was used to ensure oil solubility; a vehicle control (MHB + 1% DMSO) was included to confirm the absence of solvent-induced inhibition.

Plates were incubated at 37 °C for 24 h. After incubation, the following parameters were recorded: Visually determined MIC (vMIC), although absorbance was measured, the vMIC was confirmed as the lowest concentration with no visible turbidity, which correlated with the optical density (OD) measured at 630 nm using an mcr^®^ microplate reader (Rayto, Shenzhen, China).

Exploratory estimated MIC (eMIC): This value was derived from the non-parametric statistical analysis of the dose–response data.

The experiment was performed with technical triplicates for each concentration (n = 3). To assess the consistency of the inhibitory effect, the non-parametric Jonckheere–Terpstra test was applied to evaluate the monotonic trend across the concentration gradient. This statistical approach allowed for an exploratory estimation of the MIC by identifying the concentration where the inhibitory trend reached its maximum significance. Statistical analyses were performed using RStudio software, version: 2026.01.0+392.

### 2.7. Determination of the Fractional Inhibitory Concentration Index (FICI)

A 6 × 6 well matrix was prepared, in which the antibiotic cefepime was placed in serial horizontal dilutions across columns, and the essential oil was placed in serial vertical dilutions across rows. For this method, it is necessary to start from the MIC observed for each strain. Interpretation was performed according to the following criteria: FICI ≤ 0.5, synergy; 0.5 < FICI ≤ 1.0, additive effect; 1.0 < FICI ≤ 4.0, indifference; FICI > 4.0, antagonism. The interaction between the essential oil and cefepime was evaluated by calculating the FICI using the following formula:

$$FICI=\frac{MIC\;of\;the\;oil\;in\;combination}{MIC\;of\;the\;oil\;alone}+\frac{MIC\;of\;antibiotic\;in\;combination}{MIC\;of\;antibiotic\;alone}$$

## 3. Results

### 3.1. Susceptibility Testing

**Figure 1:**
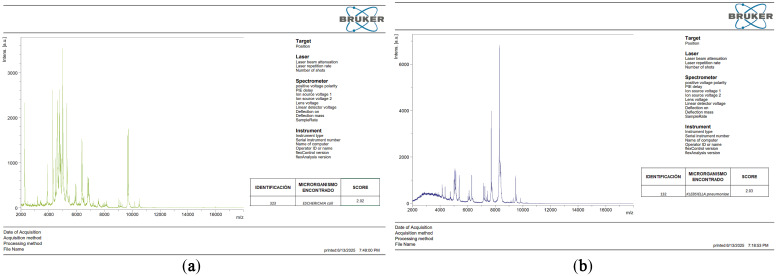
Protein spectra of the study strains (**a**) *Escherichia coli*; (**b**) *Klebsiella pneumoniae*.

The identity of the clinical isolates was confirmed using MALDI-TOF mass spectrometry, showing high-reliability scores (>2.0) for both *Escherichia coli* (strain 323) and *Klebsiella pneumoniae* (strain 132), as detailed in Table 3 and their respective protein spectra in Figure 1.

**Figure 2:**
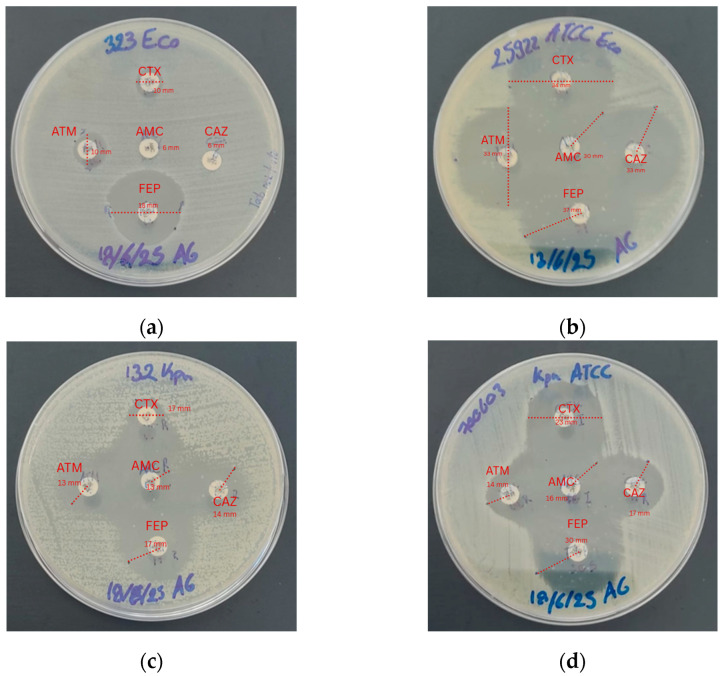
Mueller–Hinton agar plates: (**a**) antibiogram of the *E. coli* 323 study strain; (**b**) antibiogram of the *E. coli* ATCC 25922 control strain; (**c**) antibiogram of the *K. pneumoniae* 132 study strain; (**d**) antibiogram of the *K. pneumoniae* ATCC 700603 control strain.

Regarding their resistance profile, phenotypic testing via the disk diffusion method confirmed that both clinical isolates were resistant to all evaluated β-lactam antibiotics, including fourth-generation cephalosporins and monobactams (Table 4 and Figure 2).

**Figure 3:**
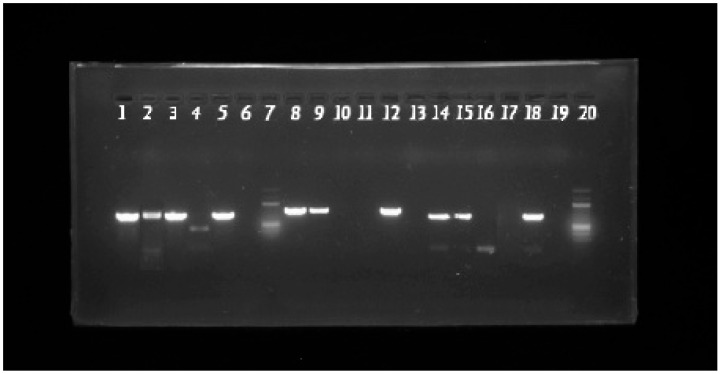
Agarose gel electrophoresis evaluating, in order: lanes 1 to 6, *K. pneumoniae* 132, *E. coli* 323, *K. pneumoniae* ATCC, *E. coli* ATCC, positive control, and negative PCR control for the *bla_SHV*; lanes 8 to 13, *K. pneumoniae* 132, *E. coli* 323, *K. pneumoniae* ATCC, *E. coli* ATCC, positive control, and negative PCR control for the *bla_TEM*; finally, lanes 14 to 19, *K. pneumoniae* 132 study strain, *E. coli* 323 study strain, *K. pneumoniae* ATCC, *E. coli* ATCC, positive control, and negative PCR control for the *bla_CTX-M*. Lanes 7 and 20 corresponded to the molecular weight marker.

To correlate this resistant phenotype with the presence of specific resistance determinants, molecular characterization was performed. As shown in Figure 3 and Table 5, PCR analysis confirmed that both the *E. coli* 323 and *K. pneumoniae* 132 strains carry the *bla_CTX-M*, *bla_TEM*, and *bla_SHV* genes. These results confirm that the observed multidrug resistance is mediated by a broad repertoire of Extended-Spectrum β-Lactamases (ESBLs). In contrast, the *E. coli* ATCC 25922 control strain was negative for all genes, while the *K. pneumoniae* ATCC 700603 served as a positive control for the *bla_SHV* gene, validating the accuracy of the genotyping.

### 3.2. Determination of the Minimum Inhibitory Concentration

Analysis for MIC determination was performed using the broth microdilution method. Cefepime was used as the antibiotic control, and ten concentrations were tested to determine the MIC. The ranges were from 2 to 1024 µg/mL, and in the case of *K. pneumoniae* 132, the MIC was evaluated up to 2048 µg/mL.

#### 3.2.1. Experimental MIC Results of Oregano Essential Oil and Cefepime

The essential oil showed an inhibitory effect against all strains, with the highest potency against *E. coli* ATCC 25922 (256 µg/mL) and the lowest potency against the *K. pneumoniae* 132 (2048 µg/mL). These values could be attributed to differences in permeability and efflux pumps. The results are shown in Table 6.

#### 3.2.2. Statistical Analysis of the Dose-Dependent Response Induced by *Origanum vulgare* Essential Oil

The estimated MIC (eMIC) values for *Origanum vulgare* essential oil against the *E. coli* 323 and *K. pneumoniae* 132 study strains and ATCC control strains are shown in Table 7. The Shapiro–Wilk test indicated that the data did not follow a normal distribution (*p* < 0.05), justifying the use of non-parametric methods.

**Figure 4:**
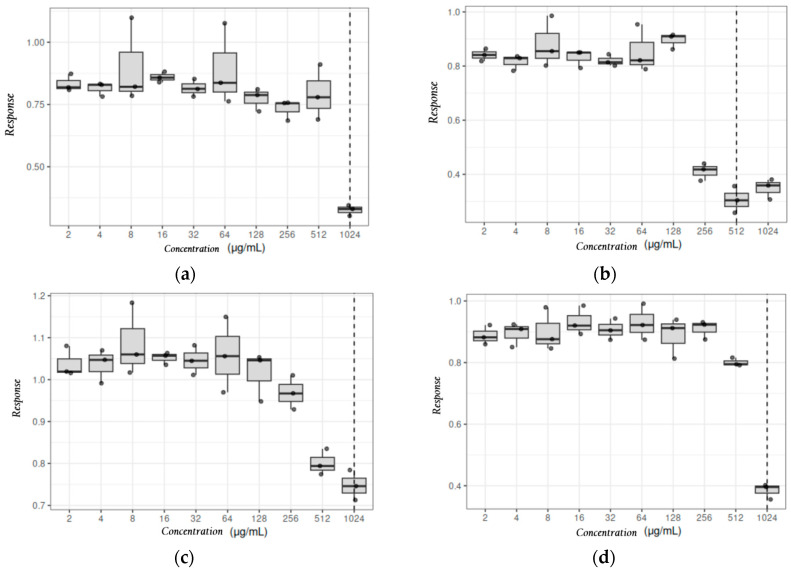
Diagrams for the exploratory determination of the MIC of *Origanum vulgare* essential oil against: (**a**) *E. coli* 323; (**b**) *E. coli* ATCC 25922 control strain; (**c**) *K. pneumoniae* 132; (**d**) *K. pneumoniae* ATCC 700603 control strain. The central line represents the median, the box represents the interquartile range, and the individual points represent experimental replicates (n = 3). The vertical dashed line indicates the exploratory MIC, defined as the concentration associated with maximum inhibition.

Given the exploratory nature of this study and the limited number of clinical isolates (n = 2), the statistical analysis focused on evaluating the consistency of the inhibitory effect across technical triplicates (n = 3) for each concentration. The Jonckheere–Terpstra test was employed to determine the existence of a significant monotonic trend in the dose–response relationship. As shown in Figure 4 and Table 7, a significant concentration-dependent inhibitory trend was observed for all strains (*p* < 0.05).

The eMIC was defined as the concentration where the maximum statistical significance of the inhibitory trend was reached. It is important to note that, in some cases, the eMIC provided a more conservative estimate than the vMIC. For instance, while the vMIC for *K. pneumoniae* 132 was 2048 µg/mL, the eMIC was established at 1024 µg/mL based on the point of maximum statistical trend stability. These statistical findings are intended to complement the experimental evidence and should be interpreted within the context of an exploratory pilot study.

### 3.3. Determination of the Fractional Inhibitory Concentration Index (FICI)

Based on the FICI results of oregano essential oil against the bacterial strains, a complete checkerboard assay was performed using a 6 × 6 matrix, and all combinations were evaluated. Six combinations showing the greatest inhibition, measured by optical density, were selected, and the most representative result is detailed in Table 8.

The results indicated a predominantly synergistic effect of the combination of oregano essential oil and cefepime against the *E. coli* 323, *E. coli* ATCC, and *K. pneumoniae* ATCC, with a more pronounced synergy observed in the *E. coli* 323 and a tendency toward additivity in the *K. pneumoniae* 132, which required significantly higher concentrations of both agents.

## 4. Discussion

Phenotypic resistance profile

The results obtained from the disk diffusion test (Kirby–Bauer) for the *E. coli* 323 showed resistance to AMC (6 mm), ATM (10 mm), FEP (18 mm), CTX (10 mm), and CAZ (6 mm), similar to the study by Kumar et al. [17], in which 65% of clinical isolates exhibited complete resistance to cephalosporins (ceftazidime, cefotaxime, and cefepime). Likewise, Rehman et al. reported resistance rates of up to 100% to cefepime and cefotaxime, with inhibition zones < 16 mm, confirming the multidrug-resistant profile of the strain evaluated in the present study [18]. In contrast, the control strain *E. coli* ATCC 25922 showed susceptibility to all evaluated antibiotics, consistent with the findings reported by Hudzicki, who indicated susceptibility to monobactams with inhibition zones equal to or greater than 30 mm in diameter [19], he inclusion of this reference strain confirmed the reliability of the phenotypic assays and served as a baseline for comparing the multidrug-resistant profile of the clinical isolates [20].

For the *K. pneumoniae* 132, reduced inhibition zones were observed: AMC (13 mm), ATM (13 mm), FEP (17 mm), CTX (17 mm), and CAZ (14 mm), in agreement with Smith et al., who established that inhibition zones below 18 mm for FEP, AMC, and ATM indicate the presence of ESBL production [21]. Similarly, Husna described that resistance to monobactams and combinations such as AMC/ATM in ESBL-producing *K. pneumoniae* strains may exceed 90% of cases, which is consistent with the results obtained in this study [22]. Regarding the control strain *K. pneumoniae* ATCC 700603, the inhibition zones were within the quality control ranges established by EUCAST/CLSI, demonstrating a typical ESBL phenotype and validating the disk diffusion method. This strain is used as an ESBL control because it carries natural resistance associated with the *bla_SHV*-18 gene [23].

Antimicrobial activity of the essential oil (MIC)

The MIC of oregano essential oil for the *E. coli* 323 was 1024 µg/mL. This value is consistent with, although slightly higher than, that reported by Burt, who obtained an average MIC of 500 µg/mL for oregano essential oil against ESBL-producing *E. coli* strains [24], This variability is attributed to differences in the chemical composition of the essential oil or to the study strains used, which may present distinct resistance profiles, resulting in greater natural or acquired susceptibility to natural compounds. For *E. coli* ATCC 25922, the obtained concentration (256 µg/mL) is consistent with the study by Khan et al., who reported an MIC of 225 µg/mL for the compound carvacrol [25]. Similarly, Stamova et al. reported a concentration of 250 µg/mL [26], supporting the validity of the results obtained in this study, as the essential oil used contains 86.74% carvacrol, providing high efficacy and confirming its antimicrobial activity through damage to the cytoplasmic membrane and alteration of cellular metabolism [24].

For the *K. pneumoniae* 132, the MIC was 2048 µg/mL. Several studies have also evaluated oregano essential oil against clinical *K. pneumoniae* strains, including those with ESBL mechanisms, and have reported MIC values ranging from 128 to 256 µg/mL [27]. Another study conducted in Bulgaria using clinical isolates reported a very broad MIC range of 390 to 12,500 µg/mL; therefore, the strain used in the present study falls within the range observed for resistant strains. Finally, for the control strain *K. pneumoniae* ATCC 700603, the observed concentration of 1024 µg/mL represented significant inhibition due to its resistant nature. In comparison, a study that also evaluated the antimicrobial activity of oregano essential oil reported an MIC of 512 µg/mL [28], while Bouhaddouda evaluated the same strain and reported MIC values of 1560 µg/mL, indicating a variable range [29].

It should be noted that, although the statistical trend analysis supported a concentration-dependent inhibitory effect starting at 1024 µg/mL, the MIC for the *K. pneumoniae* 132 was conservatively reported as 2048 µg/mL. This decision was based on the microbiological criterion of complete and consistent inhibition observed across replicates, rather than solely on statistical significance. Given the exploratory nature of the study and the intrinsic resistance of *K. pneumoniae*, this conservative approach was adopted to ensure coherence with experimental inhibition patterns.

Antimicrobial activity of cefepime (MIC)

To accurately evaluate the potential of *Origanum vulgare* essential oil as a resistance-modifying agent, it was first necessary to establish the high-level resistance baseline of the study strains against Cefepime.

The *E. coli* 323 exhibited a cefepime MIC of 64 µg/mL. This value is consistent with the study by Kumar et al. [17], in which more than 60% of clinical isolates presented a cefepime MIC of 64 µg/mL, providing clear evidence of the epidemiology of cefepime resistance in ESBL-producing strains. In addition, CLSI and EUCAST [30] indicate that MIC values above 16 µg/mL are associated with CTX-M–type enzymes and porin loss mechanisms, indicating a high resistance profile. In contrast, the cefepime MIC for *E. coli* ATCC 25922 was 0.12 µg/mL, which falls within the quality control (QC) range established by the CLSI 2025 standard, defined as 0.016 to 0.12 µg/mL; therefore, the obtained values do not exceed the established threshold.

For the *K. pneumoniae* 132, the results showed a cefepime MIC of 64 µg/mL, confirming its resistant condition. Yu et al. evaluated 110 clinical ESBL-producing *K. pneumoniae* isolates and reported that 47% of these strains exhibited resistance greater than 16 µg/mL, suggesting that such resistance levels are common in strains producing CTX-M or SHV-5 β-lactamases [30]. Patel reported that ESBL-producing *K. pneumoniae* with MIC values ranging from 2 to 8 µg/mL are associated with poor clinical response to cefepime [31]. In the case of the control strain *K. pneumoniae* ATCC 700603, the observed MIC was 1 µg/mL, which is within the QC ranges established by CLSI [16] (0.25–2 µg/mL).

Antimicrobial interaction (FICI) between *Origanum vulgare* essential oil and cefepime

The combination of oregano essential oil with cefepime exhibited a synergistic effect against *E. coli*, indicating that carvacrol and thymol enhance the action of the antibiotic by increasing outer membrane permeability and facilitating the entry of the β-lactam, a mechanism previously described in the literature [13]. In addition, recent studies have reported that essential oils rich in carvacrol can reduce the expression of β-lactamases through gene suppression and protein inhibition, thereby decreasing the MIC of antibiotics in resistant Enterobacteriaceae, which is consistent with the marked MIC reduction observed in this study [12,32].

In *K. pneumoniae* the observed effect was additive, which is consistent with investigations indicating that this species exhibits lower susceptibility to hydrophobic compounds due to its polysaccharide capsule and reduced membrane permeability [33]. Despite this, the partial reduction in the antibiotic MIC in combination suggests an adjuvant effect of the essential oil. These results, together with findings reported in recent reviews, reinforce the potential use of essential oils as enhancers of antimicrobial therapies against multidrug-resistant bacteria.

## 5. Conclusions

This study demonstrates that the evaluated clinical strains of *E. coli* and *K. pneumoniae* exhibit a high resistance phenotype to β-lactam antibiotics, consistent with the expected profiles of ESBL-producing bacteria. The use of ATCC reference strains allowed validation of the applied methodologies and reinforced the reliability of the experimental results.

*Origanum vulgare* essential oil exhibited antimicrobial activity against all evaluated strains, although differences in susceptibility between species were observed. *E. coli* showed greater sensitivity to the essential oil, whereas *K. pneumoniae* required higher concentrations to achieve inhibition, reflecting its greater intrinsic resistance. Statistical analysis supported the observed trend, indicating a concentration-dependent inhibitory trend.

The evaluation of the fractional inhibitory concentration index (FICI) revealed synergistic interactions between the essential oil and cefepime in *E. coli*, as well as additive effects in *K. pneumoniae*. These findings suggest that the essential oil, particularly due to its high content of carvacrol and thymol, may act as an adjuvant in combination approaches against resistant Enterobacteriaceae.

Overall, the findings support the antimicrobial potential of *O. vulgare* essential oil and its ability to enhance the activity of β-lactam antibiotics against ESBL-producing bacteria. However, as this was a pilot study, further investigations involving a larger number of strains, cytotoxicity assessments, and evaluation in in vivo models are required to determine safety, efficacy, and potential clinical relevance.

## Figures and Tables

**Table 1:** PCR conditions for DNA amplification.

Gene	Primer Sequence		Size	Denaturation	Hybridization	Extension	Final Extension
*bla_SHV*	SHV-F	ATGCGTTATATTCGCCTGTG	449	94 °C for 30 s	52.8 °C for 40 s	72 °C for 50 s	72 °C for 5 min
	SHV-R	TGCTTTGTTATTCGGGCCAA					
*bla_TEM*	TEM-F	AAACGCTGGTGAAAGTA	516	94 °C for 30 s	52.8 °C for 40 s	72 °C for 50 s	72 °C for 5 min
	TEM-R	AGCGATCTGTCTAT					
*bla_CTX-M*	CTX-M-F	TTTGCGATGTGCAGTACCAGTAA	172	94 °C for 30 s	52.8 °C for 40 s	72 °C for 50 s	72 °C for 5 min
	CTXM-R	CGATATCGTTGGTGGTGCCATA					

**Table 2:** Secondary metabolites of *Origanum vulgare* (oregano) essential oil.

Bioactives	Percentage
α-pinene	1.03%
α-thuyene	1.28%
β-myrcene	1.28%
α-terpinene	1.25%
δ-terpinene	1.07%
ρ-cymene	2.63%
Linalool	2.10%
β-caryophyllene	2.01%
Thymol	4.31%
Carvacrol	83.02%

Due to its hydrophobic nature, the essential oil was solubilized using 1% DMSO at a 1:1 ratio.

**Table 3:** Identification of bacterial strains.

Sample ID	Score	Corresponding Genus and Species
323	2.02	*Escherichia coli*
132	2.03	*Klebsiella pneumoniae*

**Table 4:** Results and interpretation of inhibition zone diameters in the antibiograms.

Antibiotic	*E. coli* 323	*E. coli* ATCC 25922	*K. pneumoniae* 132	*K. pneumoniae* ATCC 700603
AMC	6 mm (R)	30 mm (S)	13 mm (R)	16 mm (I)
ATM	10 mm (R)	33 mm (S)	13 mm (R)	14 mm (R)
FEP	18 mm (R)	37 mm (S)	17 mm (R)	30 mm (S)
CTX	10 mm (R)	34 mm (S)	17 mm (R)	23 mm (I)
CAZ	6 mm (R)	33 mm (S)	14 mm (R)	17 mm (R)

Note: amoxicillin/clavulanic acid (AMC), aztreonam (ATM), cefepime (FEP), cefotaxime (CTX), ceftazidime (CAZ), R (resistant), I (intermediate), S (susceptible). Interpretation was performed according to CLSI [16].

**Table 5:** PCR results and interpretation.

Bacterial Strains	*bla_SHV*	*bla_TEM*	*bla_CTX-M*
*K. pneumoniae* 132	Positive	Positive	Positive
*E. coli* 323	Positive	Positive	Positive
*K. pneumoniae* ATCC 700603	Positive	Negative	Negative
*E. coli* ATCC 25922	Negative	Negative	Negative

**Table 6:** MIC results.

Bacteria	*E. coli* 323	*E. coli* ATCC 25922	*K. pneumoniae* 132	*K. pneumoniae* ATCC 700603
Concentration µg/mL OEO	1024	256	2048	1024
Concentration µg/mL Cefepime	64	0.12	64	1

**Table 7:** Results of the Shapiro–Wilk and Jonckheere–Terpstra analyses for the exploratory MIC of *Origanum vulgare* essential oil.

Bacterial Strains	S-W	*p* Values in S-W	Interpretation of Normality	*p* Values in Jonckheere-Terpstra	Exploratory MIC (Experimental Criterion)
*E coli* 323	0.78935	4.219 × 10^−5^	Not normal	7 × 10^−4^	1024 µg/mL
*E. coli* ATCC 25922	0.77984	2.874 × 10^−5^	Not normal	0.0012	512 µg/mL
*K. pneumoniae* 132	0.87583	0.002265	Not normal	1 × 10^−4^	1024 µg/mL
*K. pneumoniae* ATCC 700603	0.64886	3.048 × 10^−7^	Not normal	0.0156	1024 µg/mL

**Table 8:** FICI results showing synergy between the essential oil and the antibiotic.

Bacterial Strains	Concentration Oregano Essential Oil µg/mL	Concentration Cefepime µg/mL	FICI
*E. coli* 323	64	8	0.188 Synergy
*E. coli* ATCC 25922	8	0.0375	0.0625 Synergy
*K. pneumoniae* 132	128	512	0.563 Addition
*K. pneumoniae* ATCC 700603	64	0.06	0.1225 Synergy

## Data Availability

The original contributions presented in this study are included in the article. Further inquiries can be directed to the corresponding author.
